# Temporary transvenous diaphragm pacing vs. standard of care for weaning from mechanical ventilation: study protocol for a randomized trial

**DOI:** 10.1186/s13063-018-3171-9

**Published:** 2019-01-17

**Authors:** Douglas Evans, Deborah Shure, Linda Clark, Gerard J. Criner, Martin Dres, Marcelo Gama de Abreu, Franco Laghi, David McDonagh, Basil Petrof, Teresa Nelson, Thomas Similowski

**Affiliations:** 1 Lungpacer Medical Incorporated, Burnaby, BC Canada; 2 Deborah Shure MD PA, Miami, FL USA; 3 Department of Thoracic Medicine and Surgery, Lewis Katz School of Medicine at Temple University, Philadelphia, PA USA; 4 Sorbonne Université, INSERM, UMRS1158 Neurophysiologie Respiratoire Expérimentale et Clinique and AP-HP, Groupe Hospitalier Pitié-Salpêtrière Charles Foix, Service de Pneumologie et Réanimation Médicale du Département R3S, Paris, France; 5 Department of Anesthesiology and Intensive Care Medicine, Pulmonary Engineering Group, University Hospital Carl Gustav Carus, Technische Universität Dresden, Dresden, Germany; 6 Division of Pulmonary and Critical Care Medicine, Hines Veterans Affairs Hospital Hines, Loyola University, Maywood, IL USA; 7 Departments of Anesthesiology and Pain Management, Neurological surgery, Neurology and Neurotherapeutics, University of Texas Southwestern Medical Center, Dallas, TX USA; 8 Meakins-Christie Laboratories, and Translational Research in Respiratory Diseases Program, McGill University Health Centre and Research Institute, Montreal, QC Canada; 9 Technomics Research, LLC, Minneapolis, MN USA; 10 Lungpacer Medical, 260 Sierra Drive, Exton, PA 19335 USA

**Keywords:** Mechanical ventilation, Weaning, Diaphragm, Ventilator-induced diaphragmatic dysfunction, Phrenic stimulation

## Abstract

**Background:**

Mechanical ventilation (MV) is a life-saving technology that restores or assists breathing. Like any treatment, MV has side effects. In some patients it can cause diaphragmatic atrophy, injury, and dysfunction (ventilator-induced diaphragmatic dysfunction, VIDD). Accumulating evidence suggests that VIDD makes weaning from MV difficult, which involves increased morbidity and mortality.

**Methods and analysis:**

This paper describes the protocol of a randomized, controlled, open-label, multicenter trial that is designed to investigate the safety and effectiveness of a novel therapy, temporary transvenous diaphragm pacing (TTVDP), to improve weaning from MV in up to 88 mechanically ventilated adult patients who have failed at least two spontaneous breathing trials over at least 7 days. Patients will be randomized (1:1) to TTVDP (treatment) or standard of care (control) groups. The primary efficacy endpoint is time to successful extubation with no reintubation within 48 h. Secondary endpoints include maximal inspiratory pressure and ultrasound-measured changes in diaphragm thickness and diaphragm thickening fraction over time. In addition, observational data will be collected and analyzed, including 30-day mortality and time to discharge from the intensive care unit and from the hospital. The hypothesis to be tested postulates that more TTVDP patients than control patients will be successfully weaned from MV within the 30 days following randomization.

**Discussion:**

This study is the first large-scale clinical trial of a novel technology (TTVDP) aimed at accelerating difficult weaning from MV. The technology tested provides the first therapy directed specifically at VIDD, an important cause of delayed weaning from MV. Its results will help delineate the place of this therapeutic approach in clinical practice and help design future studies aimed at defining the indications and benefits of TTVDP.

**Trial registration:**

ClinicalTrials.gov, NCT03096639. Registered on 30 March 2017.

**Electronic supplementary material:**

The online version of this article (10.1186/s13063-018-3171-9) contains supplementary material, which is available to authorized users.

## Background

Mechanical ventilation (MV) is a life-saving technology routinely administered in intensive care units (ICUs). In the USA, hospitalization involving endotracheal intubation and MV increased 56% between 1997 and 2011 [1]. In 2011, MV using tracheal intubation was the third most common procedure performed in this country [1]. The proportion of ICU beds occupied by patients requiring MV on a daily basis ranges from 20.7 to 38.9% [2]. The projected costs to care for the estimated 625,000 US patients requiring prolonged MV in 2020 are expected to be $64 billion [3].

Like all active treatments, MV can be associated with several adverse effects. They include lung injury [4, 5], pulmonary infections [6], and the more recently recognized ventilator-induced diaphragmatic dysfunction (VIDD) [7–9]. VIDD most probably proceeds from MV-associated diaphragmatic inactivity or unloading, possibly aggravated by various ICU-related aggressions (sepsis, etc.). VIDD is characterized by severe muscle fiber atrophy and an array of muscle lesions [10]. The onset of diaphragm atrophy under MV is rapid, with abnormalities becoming visible histologically and functionally after only a few hours [11, 12].

Accumulating evidence suggests that VIDD is associated with delayed liberation from MV, longer ICU stays, and a higher risk of complications during the ICU stay [13]. In patients who are difficult to wean from MV, mortality increases with each additional day spent on the ventilator [14]. Animal studies suggest that keeping the diaphragm active during MV reduces VIDD development. This potential to prevent VIDD [15] remains to be established in human clinical trials, and no clinically validated cure or treatment for VIDD currently exists. Of interest, limited evidence supports the putative usefulness of inspiratory muscle training to accelerate weaning from MV in patients requiring prolonged mechanical ventilation in whom VIDD may be already present [16, 17].

Inspiratory muscle training is difficult to administer and standardize. It has therefore been proposed that pacing the diaphragm through phrenic nerve stimulation could be a solution to counteract VIDD by restoring diaphragmatic muscle mass and function, hence improving the weaning outcome [18–20]. The ability of diaphragm pacing to recondition atrophic diaphragms has long been demonstrated by studies conducted with surgically implanted stimulators in quadriplegic patients with high cervical spinal cord lesions (see recent review in [21]). In the context of “acute” MV, animal studies have shown that diaphragm pacing when superimposed on MV can mitigate the development of VIDD [22, 23]. Preliminary insights into the mechanisms responsible for this effect have also been obtained [24–26].

An absolute prerequisite for the clinical application of diaphragm pacing to overcome VIDD in the ICU is to make phrenic stimulation feasible at the bedside. Surgical techniques cannot be considered in this context. Magnetic stimulation has been proposed, but it is cumbersome [27]. Transvenous phrenic stimulation has been previously assessed in various animal models (first mentioned in reference [28]), albeit in a clinically impractical form. Recent data have shown that transvenous phrenic stimulation could be achieved in humans on a permanent basis to treat central sleep apneas [29] by means of a permanent implant. The Lungpacer LIVE® Catheter provides a technological solution to achieve temporary transvenous diaphragm pacing (TTVDP) in ICU patients through a minimally invasive bedside procedure. This system includes a proprietary intravenous multi-electrode stimulating catheter which can also deliver intravenous fluids. In the current state of the technology, a catheter is inserted in the left subclavian vein in the same way as used for a standard subclavian line. The catheter-borne electrodes provide stimulation of the left phrenic nerve as it enters the thorax posterior to the subclavian vein and stimulation of the right phrenic nerve at the level of the atriocaval junction. A first-in-human study showed that this technology delivered safe and effective diaphragm pacing [30].

The Lungpacer RESCUE 2 study (NCT03096639) tests the main hypothesis that TTVDP used in ICU patients who are difficult to wean from MV will improve the weaning outcome. Specifically, RESCUE 2 is a prospective, randomized, controlled, open-label, multicenter study with a primary objective to evaluate the efficacy of TTVDP in terms of the probability of successful weaning on day 30 using a competing risks survival model. The primary endpoint is the time to successful extubation with no reintubation within 48 h (or, in tracheotomized patients, the time to successful 24-h separation from the ventilator with no replacement on MV within 48 h). Moreover, RESCUE 2 evaluates the safety of TTVDP in the clinical context of weaning from MV.

**Fig. 1:**
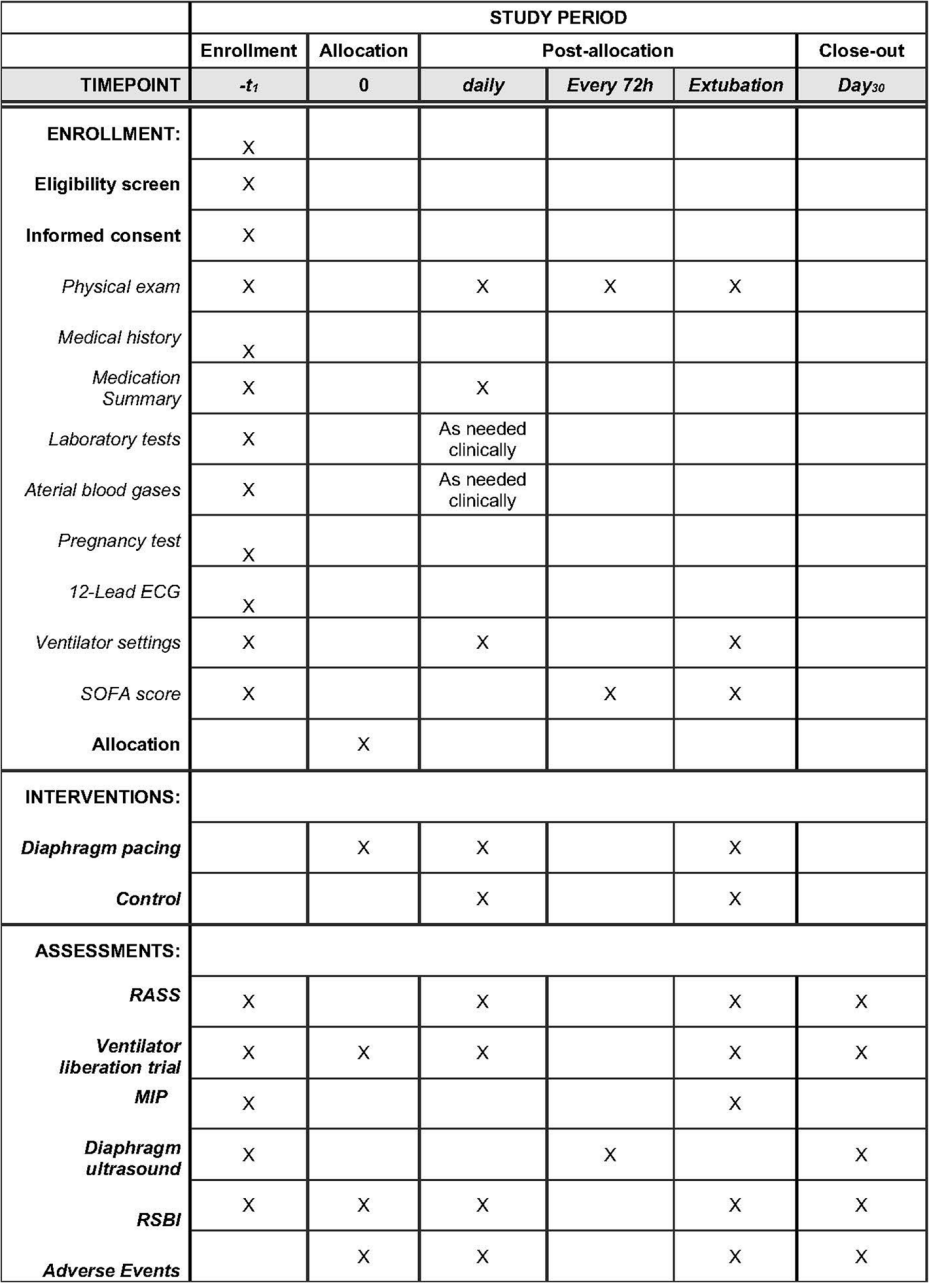
SPIRIT figure describing study procedures and assessments. *SOFA* sequential organ failure assessment, *RASS* Richmond Agitation-Sedation Scale, *MIP* maximal inspiratory pressure, *RSBI* rapid shallow breathing index

The RESCUE 2 protocol (Revision F, as of August 31, 2018) as described in the present manuscript conforms to the Standard Protocol Items: Recommendations for Interventional Trials (SPIRIT) guidelines (http://www.spirit-statement.org/) (see Fig. 1 and Additional file 1).

## Methods

### Study setting

This prospective, multicenter, randomized, controlled, open-label interventional study evaluates weaning from MV after TTVDP (treatment) or standard of care (control). In the treatment arm, in addition to standard of care, the study involves the percutaneous insertion of the LIVE® Catheter using the left subclavian approach into the superior vena cava, the capture of the left and right phrenic nerves by stimulation electrodes, and repeated daily sessions of diaphragm pacing conducted until liberation from MV. In the control arm, the study involves standard of care only, without LIVE® Catheter insertion or any kind of sham treatment (open-label design). In the trial patients will be followed until successful liberation from MV or for 30 days, whichever comes first. All study patients will be monitored for 48 h after extubation (or disconnection from MV for tracheotomized patients) and removal of the LIVE® Catheter. If a study patient is extubated on day 29 or 30, the 48 h follow-up will extend study participation out to a maximum of 32 days after randomization. All study patients will be followed to day 30 for all serious adverse events (SAEs), ICU and hospital admissions, discharges, and mortality.

Notwithstanding ulterior amendments, this study will be conducted in 10 European centers in France and Germany. These university and academic institutions are evaluating patients in a variety of ICUs as well as specific weaning units. All hospitals have established experienced research teams skilled at managing critical care patients on MV. The full list of the participating centers is provided in the Appendix.

### Eligibility criteria

Prior to randomization, all study participants will undergo screening to determine eligibility. Screening evaluations will include physical examination, medical history, medication history, vital signs, weight, electrocardiography (ECG), blood tests, and arterial blood gas assessments.

Study participants will be included if they:

- Are at least 18 years old
- And have been on “invasive” MV for at least 7 days (patients can be either intubated or tracheotomized; the protocol does not involve any interference with local procedures regarding the decision and timing of tracheotomy)
- And have failed at least two attempts at ventilator liberation, henceforth referred to as “ventilator liberation trials” (VLTs); one VLT must be the study-specific VLT (see subsequent sections); self-extubation or accidental extubation with subsequent reintubation within 48 h is considered a failed VLT.

Patients cannot be included if they:

- Are currently on extracorporeal membrane oxygenation
- Have failed weaning from MV because of current hypervolemia
- Have known anatomical features preventing catheterization of the left subclavian vein
- Have a history of congenital heart disease
- Have clinically overt congestive heart failure
- Are currently being treated with a neuromuscular blockade
- Have pre-existing neuromuscular diseases potentially affecting respiratory muscles
- Have pleural effusions occupying greater than one third of the pleural space on either side
- Have a body mass index ≥40 kg/m^2^
- Have known or suspected phrenic nerve paralysis
- Have any electrical device (implanted or external) potentially prone to interaction with or interference from the temporary transvenous diaphragm pacing system, including neurological pacing/stimulator devices, cardiac pacemakers, and defibrillators
- Have been diagnosed with bacteremia (blood cultures must be negative for 48 h to consider inclusion)
- Have current hemodynamic instability, sepsis, or septic shock
- Are terminally ill with 6 months or less life expectancy or not committed to full care
- Are known or suspected to be pregnant or lactating
- Are actively participating in another clinical trial
- Cannot consent to participate or belong to vulnerable populations.

### Randomization

Patient allocation to the control or TTVDP group will result from a 1:1 randomization with variable block size within each study center using the electronic data capture (EDC) system *Syncrony™* (Version 2018.01.02, Syntactx Technologies, New York, NY, USA). The randomization feature of Syncrony EDC was designed to utilize a randomization table created by an independent biostatistician using SAS 9.4 (SAS Institute Inc., Cary, NC, USA) according to the requirements in the study protocol. To ensure allocation concealment, the next randomization is unknown to the study center until the time at which a patient has signed informed consent, unique patient-identifying information is entered into the EDC, and the randomization button is pressed by the coordinator.

### Masking

This is an open-label study during which neither patients nor investigators will be blinded to treatment arm, primary outcome, or secondary outcomes.

Diaphragmatic ultrasound studies will be performed to provide diaphragm muscle characteristics as secondary outcomes in a subset of the participating sites. The analysis will be centralized at a core ultrasound laboratory where ultrasound reviewers will be blinded to the treatment arm (single blind).

### Management of control patients

Patients randomized to the control group will not receive TTVDP and will continue to be treated with the standard of care for patients who have difficulty being weaned from MV according to the procedures in effect at the study sites. The patients will undergo a daily weaning readiness assessment which, if positive, will be followed by a protocol-specific VLT conducted without pressure support and without positive end-expiratory pressure [31].

In addition to routine tests performed according to each site’s procedures, the following assessments will be performed:

- Maximal inspiratory pressure (MIP) according to the “coached version” of the unidirectional valve method described by Marini et al. [32] and Caruso et al. [33]; identical measurement procedures and devices will be used at all centers; MIP will be measured at time of enrollment, prior to extubation, and optionally every 7 days
- Rapid shallow breathing index (RSBI), calculated as the ratio of respiratory rate to tidal volume in liters [34], as part of the daily protocol-specific VLT or, when impossible, under MV set to an assisted breathing mode
- Sequential organ failure assessment (SOFA) score [35, 36] at the time of enrollment and every 72 h until removal of the TTVDP
- Diaphragmatic ultrasound measurements on day of randomization, every 3 days, and on the day of extubation, to measure diaphragm thickness and thickening fraction (TFdi) and maximal diaphragmatic excursion (EXdi) [37]; these imaging measurements will be conducted according to a protocol-specific procedure, and the designated study site staff members will have received training and be qualified to conduct these imaging studies; a blinded review of all diaphragmatic ultrasound images will be performed at a core laboratory.

### Management of treatment patients

Participants randomized to the diaphragm pacing group will receive TTVDP but will otherwise be managed exactly as the patients randomized to the control group.

### Intervention protocol

#### Device description

**Fig. 2:**
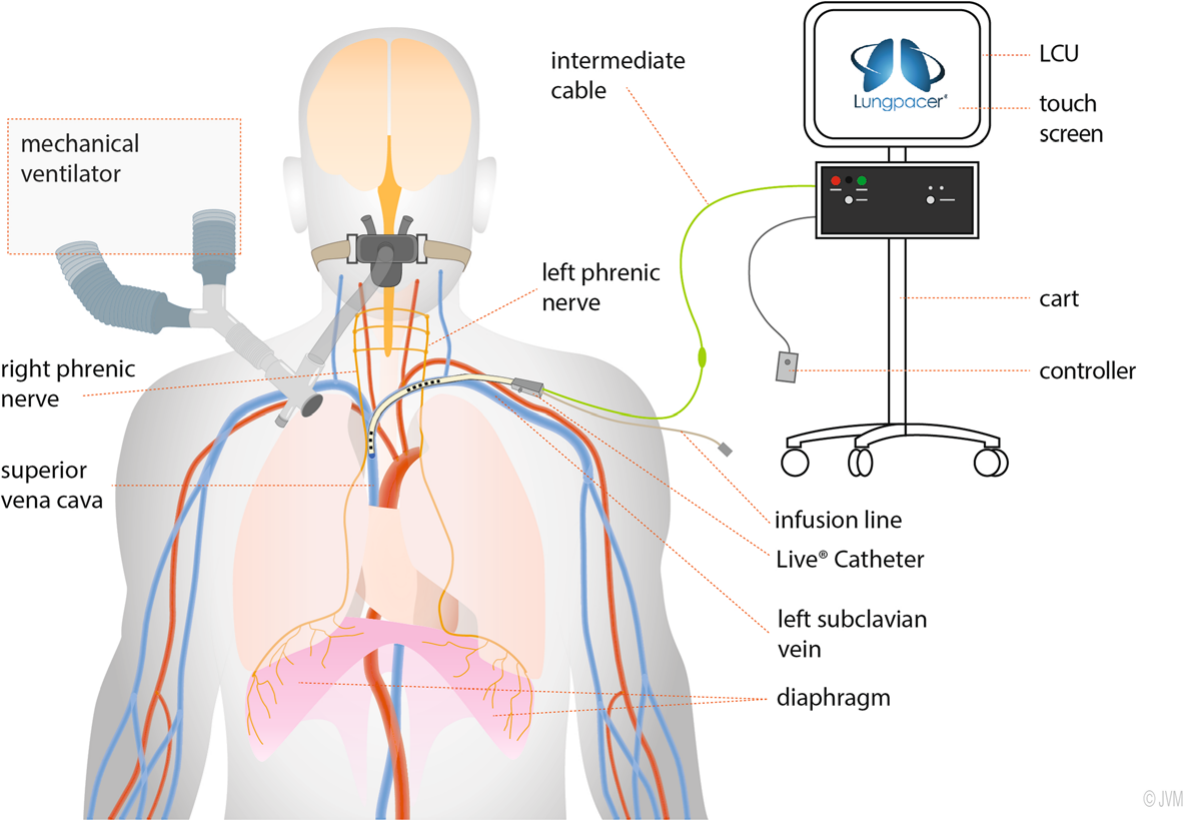
Schematic representation of the Lungpacer LIVE® Catheter system providing temporary transvenous phrenic stimulation for diaphragm pacing (*LCU* Lungpacer Control Unit). Credit to Mr. Jean Allard

The temporary transvenous diaphragm pacing system (TTVDPS, Lungpacer Medical Incorporated, Burnaby, BC, Canada) comprises:

- The LIVE® Catheter, a sterile, size 9.5 French, single-use disposable catheter resembling a typical polyurethane central venous catheter, further comprising two arrays of electrodes targeting the left (proximal array) and right (distal array) phrenic nerves, a single fluid lumen, and a primary cable containing electrical leads which terminate at an electrical connector
- A cart-mounted Lungpacer Control Unit (LCU) including a touch screen user interface
- An intermediary cable connecting the primary cable and control unit
- A handheld controller used to deliver electrical impulses as set by the physician on the controller unit (Fig. 2).

The pulses have an intensity of up to 13.5 mA and a duration of 200–300 μs; they can be delivered with a frequency of 4 Hz for the mapping procedure (see the next section) and 15 Hz for the diaphragm pacing therapy itself.

#### Diaphragm pacing therapy

In the treatment group, the LIVE® Catheter will be inserted in the left subclavian vein using the Seldinger technique. Correct positioning of the catheter will be verified per the standard of care in each institution. A mapping procedure will be conducted before each therapy session to ensure adequate capture of both phrenic nerves and to determine the stimulation thresholds at which visible or manually palpable diaphragm contractions appear in response to the electrical impulses. The intensity will then be increased up to the maximal level tolerated by the patient. Therapy sessions will consist of 4 sets of 10 stimulations manually administered by a trained team member. The stimulation sets will be separated by a brief rest period as needed. Three therapy sessions (totaling 120 stimulations) will be conducted daily (morning, afternoon, and evening, with no less than 3 h between sessions).

#### Discontinuation of therapy

The diaphragm pacing therapy sessions will be stopped when the physician determines that the patient has successfully passed the protocol-specific VLT and can be separated from the ventilator. If discontinuation of therapy occurs before the 30-day study completion, the LIVE® Catheter will not be removed until the patient has met the weaning success criterion (48 h off the ventilator).

### Outcomes

The primary efficacy endpoint is time to successful extubation with no reintubation within 48 h. In tracheotomized patients, the primary efficacy endpoint is time to successful 24-h separation from the ventilator with no reconnection within the following 48 h.

The primary statistical analysis will model the time to event of interest, i.e., successful weaning, in the presence of competing events which include death or withdrawal of life support. The endpoint is evaluated in a competing risks survival model. In addition, withdrawal of life support and cessation of weaning in the ICU with discharge from the ICU to a specialized weaning facility by day 30 will be reported.

The secondary efficacy endpoints are:

- Time to first successful VLT after randomization
- Difference in MIP changes between study groups from randomization to successful weaning or on day 30, whichever comes first
- Difference in changes in diaphragmatic thickness (centralized blinded ultrasound evaluation) between study groups from randomization to successful weaning or on day 30, whichever comes first
- Difference in changes in diaphragm thickening fraction (centralized blinded ultrasound evaluation) between study groups from randomization to successful weaning or on day 30, whichever comes first
- Daily measurement of RSBI.

The safety endpoints will include characterizing the adverse event profile and comparing the nature and frequency of adverse events in patients randomized to TTVDP vs. control (standard of care) treatment. This safety assessment and the study risk management plan are described in detail within the study protocol.

Of note, additional observational data points will be collected, including (but not limited to) time to extubation, time to reintubation, time to ICU discharge, time to hospital discharge, and 30-day mortality from randomization.

### Recruitment capacity and consent

We have calculated a sample size of 88 patients randomized 1:1 with 44 patients anticipated in each group (see below), to be enrolled over 14 months. Before any study-related activities begin, each patient (or the patient’s legally authorized representative) will be provided details of the study by an investigator and asked if they are interested in participating in this study. If they agree they will be asked to sign the informed consent form. Participation is voluntary and does not affect standard treatment in any way. Study patients may decide to end their participation in the study at any time.

### Population size

The sample size needed to show a statistically significant difference between treatment depends on the proportion of patients successfully weaned and the proportion succumbing to a competing risk prior to successful weaning by day 30 (see the previous section “Outcomes”). Since TTVDP is a novel, first-of-its-kind therapy both in general and in the population of patients experiencing difficult weaning from MV, no estimates of the effect size or the competing events were available. Consequently, a sample of convenience was used to allow enrollment and randomization of up to 88 study patients (44 per arm).

Using a competing risks model with a log rank test (PASS 14, NCSS Statistical Software, LLC, Kaysville, UT, USA), this population size gives the study a 70% power to find a statistically significant difference in the event of a dramatically large treatment effect (i.e., approximately 50% of patients weaned with 34% experiencing one of the competing risks in the control group, vs. 80% weaned with 11% experiencing one of the competing risks in the TTVDP group).

As a result, the totality of evidence is expected to indicate a trend in favor of TTVDP (with an adverse event profile not significantly different between the groups) rather than to show definitive superiority.

Of note, Revision F of the protocol as described in this manuscript is being amended to account for a higher than expected number of withdrawals due to inability to insert the guidewire: the target size population will be brought from 88 to 110, with the possibility for sites to include two roll-in subjects for training purposes. Accordingly, the number of participating centers will be increased from 14 to 22.

### Data processing

#### Procedures

The RESCUE 2 study complies with the Declaration of Helsinki and will be conducted in accordance with the principles of Good Clinical Practice. Standardized procedures and specific training will be required as investigators select participants, obtain informed consent, and perform protocol-specific actions and measurements, including, for example, VLTs, MIP measurements, diaphragmatic ultrasound imaging, adverse event reporting, data recording, and statistical analyses.

#### Data management

Data management will be assigned to a dedicated contract research organization (CRO), namely Syntactx Ltd., Brussels, Belgium. The Syncrony™ web-based EDC database manufactured by Syntactx Technologies (New York, NY, USA) is being used to record and manage study data and to provide an audit trail. The electronic clinical data system was validated prior to first use in accordance with the CRO’s standard operating procedures. Electronic case report form (eCRF) completion guidelines and instructions for electronic data entry were developed in conjunction with the sponsor and the CRO. The EDC data can be exported to various file formats for statistical analysis. Patient data are fully anonymized, and all study numbers to identify study patients are stored separately and securely with limited access by the researchers and monitors. All data queries will be resolved before the database is locked for analysis at the end of the trial.

### Safety

Extensive preclinical testing of the LIVE® Catheter system has been conducted in compliance with harmonized standards and international medical device directives. Prior to human testing, bench and animal testing ensured that the device met the minimum design requirements for various measures, including (but not limited to) system integrity, biocompatibility, sterility, and packaging/storage integrity. The safety of the system was initially tested in a first-in-human study [30], and this RESCUE 2 study will optimize the risk/benefit balance by (1) excluding patients with conditions thought to increase procedural risks or to decrease the probability of efficacy; (2) selecting experienced investigators and experienced clinical research teams; (3) training investigators on the protocol implementation methods; and (4) utilizing experienced medical professionals to continuously monitor study patients during the catheter placement procedures and during therapy sessions.

The principal investigator at each participating center is responsible for overseeing the timely and adequate reporting of adverse events, adverse device effects, and device deficiencies, per the corresponding definitions provided in the study protocol. Investigators will report SAEs and serious adverse device effects to the study sponsor within 24 h of their first knowledge of the event. Each reported adverse event will be qualified as anticipated (a list of anticipated events exists in the study protocol) or unanticipated. The event will be assessed by the investigator for its relationship to the device or procedure (not related, unknown, possible, probable, definite) and for its severity. Adverse events will be reported on the eCRF as soon as practically possible. In case of death, the relationship of death to the medical device and/or the study will be documented as precisely as possible. The study is overseen by a Clinical Events Committee as well as a Data and Safety Monitoring Board.

The study sponsor will report SAEs, serious adverse device effects, and device deficiencies that might have led to an SAE to the national competent authorities of each country where the study is taking place.

### Statistical analysis

After the database is audited and locked, the statistical analysis will be conducted by a statistician independent of the sponsor and the CRO. The statistical plan described in the study protocol includes details about handling missing data, assessing comparability of randomized groups, and evaluating the pooled data collected at the various centers.

The data from the study will only be analyzed once at the end of the study, and no formal statistical stopping rules allow for the early termination of this study.

The analysis groups include the (1) intent-to-treat (ITT) population, defined as all patients who were randomized with a study group defined by the randomization; (2) modified intent-to-treat (mITT) population, defined as the complete control group and a subset of the ITT therapy group with successful cannulation of the left subclavian vein; and (3) per protocol (PP) population, i.e., a subset of the mITT population defined as those patients who completed at least 75% of the protocol-required stimulations in at least one phrenic nerve. The sidedness of the phrenic nerve stimulation can change from left to right or can occur in both phrenic nerves.

Descriptive statistics for continuous variables will include the number of observations, mean, standard deviation, minimum, median, and maximum. Descriptive statistics for categorical variables will include the number and percent in each category out of the total number of observed responses. The *p* values will be considered statistically significant if the two-sided *p* value is ≤0.05 unless otherwise specified. When the arithmetic mean is found not to be an appropriate measure of central tendency, alternative statistics will be considered (e.g., median, interquartile range). When the distribution of a variable does not support the use of parametric statistics, nonparametric approaches or data transformations may be implemented. If data transformations are used, they will be specified in the final clinical study report.

Regarding the primary efficacy endpoint (time to successful extubation without reintubation in the next 48 h), the null hypothesis states the cumulative incidence by day 30 for successful weaning is the same in patients randomized to treatment or control groups. The primary statistical analysis will be conducted on the mITT population and will model the time to the event of interest in the presence of competing events which include death and withdrawal of life support. In addition, cessation of weaning in the ICU with discharge from ICU to a specialized weaning facility will be evaluated. Patients who do not meet one of the endpoints by the end of their study follow-up will be censored at the time of study discontinuation or completion. The primary statistical model will utilize the cumulative incidence function. The event of interest, successful weaning, will be compared between randomized groups using Gray’s test. The probabilities will be summarized for successful weaning and each competing event for the duration of the study. The 95% confidence intervals will be provided for the probabilities at 30 days. In addition, descriptive statistics will be prepared by randomized group for the time to successful weaning in days.

All ITT patients will be included in the primary safety endpoint analysis. Fisher’s exact test will be used to compare the number of patients with an event or in a category of events out of the total number of patients randomized between groups as a tool to understand the adverse event profile associated with TTVDP compared to control.

### Monitoring, auditing, and strategy to improve adherence to the protocol

Adverse events are reviewed by a medical monitor to determine whether the adverse event is serious and to identify the SAEs that are unanticipated. SAEs are further adjudicated by an independent Clinical Events Committee with respect to relationship to the device or procedure. Additionally, a Data and Safety Monitoring Board will periodically review the study data and may recommend early termination of the trial for safety concerns. A routine audit was conducted of the CRO at initiation of the study, and a second will be conducted before the completion.

Data management, monitoring, and auditing will be provided by an independent CRO. eCRFs will be reviewed by a clinical monitor appointed by the CRO who will determine acceptability with respect to data integrity, completeness, and accuracy. Queries will be generated for omissions, reasons for missing data, corrections, and clarifications. The monitors will perform source data verification and a review of informed consent processes and study procedures. In addition to the CRO, the study sponsor may conduct announced or unannounced monitoring visits or audits of the investigational sites.

Sponsor representatives are responsible for ongoing site support which involves any retraining should a pattern emerge regarding adherence to the protocol. Investigator meetings are held once a year, and routine newsletters are provided to the investigative sites. Mini pocket protocols have been provided to the sites as well as training tools on common procedures. Sponsor representatives also conduct reviews of patient cases with the site investigators to address any concerns. Through the monitoring process the sponsor is notified of any protocol deviations and evaluates the reason behind the deviation and decides if a change to the protocol is needed or if corrective action is needed to prevent these from reoccurring. Retraining is conducted by the sponsor if warranted as the sponsor representatives engage with each investigative site.

### Ethics, amendments, and dissemination

The RESCUE 2 study has been ethically approved by competent institutional bodies in the two participating countries. In France, a global authorization relevant for all study sites has been granted by the Comité de Protection des Personnes Sud-Est VI, Clermont-Ferrand, France (decision number dated November 9, 2017) and by the competent authority (Agence Nationale de Sécurité du Médicament et des produits de santé) on June 3,2017. In Germany, the study has been approved by the central Ethics Committee RWTH Aachen (August 14, 2017), which is relevant for each participating center, and the competent authority (Bundesinstitut fur Arzneimittel und Medizinprodukte (BfArM)) on September 1, 2017 .

The RESCUE 2 study is publicly registered at the ClinicalTrials.gov database (NCT03096639) and the European Database on Medical Devices (Eudamed CIV-17-06-020004).

After data review, resolution of all queries, and completion of the database audit trail according to the protocol data management plan, the database will be locked and the preplanned statistical analysis conducted. Results will be presented at national and international conferences and submitted to peer-reviewed journals. Post-publication press releases are possible.

### Substudies

Participating centers are allowed to conduct substudies, provided that (1) no interference with the primary protocol occurs; (2) approval by the local institutional review board is obtained; and (3) the steering committee and the sponsor accept the proposal according to its originality, feasibility, and importance. Currently, substudies with electrical impedance tomography, magnetic phrenic nerve stimulation, respiratory system mechanics and variables, and transpulmonary pressure measurements are under evaluation. Publication of substudies, in any form, will not occur until the results of the primary study have been published.

## Discussion

The RESCUE 2 study will be the first controlled study of temporary transvenous diaphragm pacing (TTVDP) in patients who are difficult to wean from MV, a highly relevant clinical challenge. It will be the first study to specifically address VIDD as the cause of weaning failure and to test the hypothesis that TTVDP can accelerate difficult weaning from MV. This hypothesis is supported by the likely pathophysiological role of ICU-acquired diaphragmatic dysfunction in difficult-to-wean patients and by the known capacity of diaphragm pacing to correct diaphragmatic disuse atrophy in long-term MV-dependent patients with quadriplegia.

### Position of the study in the landscape of weaning therapeutic trials

Accelerating weaning from MV has been a major preoccupation of ICU management in general for several decades, and it is a constant focus of intensivists regarding each of their mechanically ventilated patients [38]. Protocolizing the weaning process can reduce the duration of mechanical ventilation [39] with automated weaning as a possible further step [40]. In the particular context of difficult weaning, therapeutic approaches correcting ICU complications can be useful (e.g., correction of delirium [41], correction of anemia [42, 43], alleviation of anxiety [44]), as can be the reduction of the load imposed on the inspiratory muscles (augmentation of cough [45], reduction in work of breathing through optimization of posture [46], and inhalation of low viscosity gas mixtures [47]). Correcting weaning-induced cardiovascular compromise, a major pathophysiological determinant of weaning failure in specific patients, has also been the object of several studies (brain natriuretic peptide-guided fluid management [48, 49], vasodilators [50, 51], inotropic drugs [52]).

In recent years, diaphragmatic dysfunction has emerged as a likely determinant of difficult weaning from MV [13, 53], and observational data have suggested that the recovery of inspiratory muscle force could favor weaning success [54]. This leads to the idea that restoring diaphragm strength could be clinically useful. Results of small studies suggest that diaphragm strength—a physiological outcome—can be improved in patients who are difficult to wean (with theophylline [55] or with inspiratory muscle training [56]). Beyond that, preliminary data indicate that inspiratory muscle training can facilitate weaning from MV [17], although heterogeneous results imply caution [16, 57, 58].

In this context, RESCUE 2 will test a technology that is completely novel and that will be used therapeutically for the first time. A major advantage of this approach is that, contrary to inspiratory muscle training, it does not require patient cooperation or motivation. The study will be larger in size than most of the studies that have previously attempted to demonstrate a benefit of a therapeutic approach in difficult-to-wean ICU patients. Because of the “first-of-its-kind” nature of the therapy, it was not possible to power the study according to previous data and a sample of convenience was chosen, which does constitute a methodological limitation. However, note that many published studies validating indicators predictive of weaning success or failure have been conducted in smaller or similar samples [34, 59, 60]. This suggests that a sample of this size should be sufficient to detect differences between success and failure among patients.

### Strengths, limitations, and uncertainties

#### Masking

A sham design has not been retained for the RESCUE 2 study because of the risks inherent to central venous catheterization. Therefore, the RESCUE 2 protocol does not require the placement of a central line in a control patient if this is not decided by the clinicians in charge of this patient in the context of routine care. The study is therefore controlled but not blinded, with the ensuing requirement for caution when interpreting the data. To limit the corresponding risk of bias, precautions have been taken to minimize the risk of discrepancies between the treatment and the control groups, notably regarding the inclusion process (e.g., requirement for a standardized VLT before inclusion in the study). Also, ultrasound diaphragm measurements will be evaluated by blinded operators at a core laboratory, which will allow one to credibly assess the ability of TTVDP to restore diaphragmatic mass and contractility in the study population, irrespective of the main outcome. Of note, blinded trials of medical devices are the exception rather than the rule.

#### Phrenic stimulation regimen

In the absence of an animal model relevant to difficult weaning from MV in humans, it was not possible to determine the optimal phrenic stimulation regimen in preclinical studies. The chosen regimen therefore derives from training protocols used in rehabilitation, such as the inspiratory muscle training study conducted by Martin et al. [17]. Of note, in RESCUE 2 phrenic stimulation is delivered with 200–300 μs pulses at 15 Hz, which is in the range of the parameters used to successfully mitigate VIDD in animal studies [22, 23] and known to provoke fused diaphragmatic contractions in humans [27].

#### Transposability of diaphragm pacing data in quadriplegics

The experience gained worldwide since the 1970s with diaphragm pacing attests to its efficiency at reconditioning the atrophied diaphragm in humans [21]. Diaphragm pacing-induced reconditioning in this context is sufficient to provide full breathing autonomy [21]. Success has been observed after years of disuse, namely in patients with extremely atrophic diaphragms. Yet ICU-acquired diaphragmatic dysfunction does not stem from disuse only, but most probably from an array of physiologic aggressions of various natures, including sepsis [61]. This could interfere with the anabolic effects of diaphragm pacing in a manner that is difficult to predict. In this regard, it is important to note that phrenic stimulation in patients who are difficult to wean from MV is not intended to provide full ventilatory support as in the case of quadriplegic patients, but only serves a reconditioning purpose. With this in mind, the ability of diaphragm pacing to mitigate “pure” VIDD in a preventive manner [22, 23] is reassuring.

#### Risk-benefit balance

The risks induced by TTVDP fall into two categories. The risks of central venous catheterization via the subclavian route are well known. Evaluating the corresponding risk-benefit balance is routinely done in ICU practice. Of note, a transjugular approach may reduce some of the risks associated with central venous catheterization: this technology is currently not available, but it is under development. The risks inherent to diaphragm pacing itself are less well defined. In quadriplegic patients, clinical experience and the literature suggest that these risks are minimal [21], particularly regarding the issue of phrenic nerve damage. Yet in the context of amyotrophic lateral sclerosis, direct diaphragm pacing has proven deleterious [62, 63], which indicates that caution is required. The presence of sepsis is associated with diaphragmatic structural lesions that are more severe in the presence than in the absence of diaphragm contractions [64, 65], in part because of an increased local production of inflammatory mediators [66]. Sepsis is among the study’s exclusion criteria, and as such a worsening diaphragmatic dysfunction through sepsis-related mechanisms appears relatively unlikely. Of note, short-term human uses of the LIVE® Catheter have not unveiled significant safety concerns [30]. Finally on this point, it should be kept in mind that the potential benefits of TTVDP in patients who are difficult to wean from MV are major. Indeed, in the context of difficult weaning from MV, each day spent on the ventilator is associated with additional morbidity and mortality [14].

#### Generalizability of the expected results

Inclusion in the RESCUE 2 study is, purposefully, not very selective, the main entry criterion being clinical (“difficult weaning from mechanical ventilation”) rather than mechanistic (“physiologically documented VIDD”). The RESCUE 2 study population will therefore include patients in whom difficult weaning does not necessarily primarily proceed from VIDD (or, more generally, ICU-acquired diaphragmatic dysfunction). Of note however, several known causes of difficult weaning like hypervolemia, overt congestive heart failure, and abundant pleural effusion do appear to constitute exclusion criteria. This recruitment strategy does carry the risk of diluting the effect of the therapy in what is already a heterogeneous population, and this could be considered a weakness of the study. We however consider this to actually be a strength. Indeed, there is currently no easy-to-use validated approach to identify diaphragmatic dysfunction in ICU patients. The need for such identification before considering TTVDP would set a stringent limitation to the use of the technique in clinical practice. Accordingly, it appears very important to test the hypothesis that TTVDP can be used to promote weaning in patients not specifically assessed for diaphragmatic dysfunction. This is clinically reasonable insofar as the proportion of patients having received MV for 7 days or more who suffer from diaphragmatic dysfunction (and therefore exhibit a load-capacity imbalance) is very high [53]: correcting VIDD can therefore help overcome weaning difficulties even in the presence of other contributing factors. As a result, a positive outcome in spite of the nonselective approach chosen for the study would give it major clinical relevance.

In contrast, a negative outcome would not mean that the therapy is not useful in more specifically selected patients: in this view, the results of the blinded ultrasound evaluation of the effects of TTVDP on diaphragm mass and contractility will be of great use and importance. Of note in this regard, diaphragm pacing could improve the ability of patients to be weaned from MV not only through improved diaphragm function but also through other mechanisms such as improved lung mechanics and gas exchange through enhancement of lower lobes aeration [67] and positive hemodynamic effects [68].

The RESCUE 2 study is the first controlled study of TTVDP in patients who are difficult to wean from MV, a highly relevant clinical challenge. It also the first study designed to specifically address VIDD as the cause of weaning failure. Its results will help delineate the place of this therapeutic approach in clinical practice and help design future studies aimed at defining the indications and benefits of TTVDP.

## Trial status

The first patient was enrolled on September 29, 2017, and 31 patients were enrolled as of July 2018. Enrollment is expected to be completed in September 2019.

The protocol described in this study is “Revision F”. This version is in force at the time of submission but is in the process of being amended to “Revision K”. Among the main changes are an augmented population size (see the section on “Population size”), an augmented number of centers, and follow-up of patients when transferred to an outside facility to ascertain weaning success or failure by day 30 (thereby removing this event from the list of the competing risk events).

### Additional file

Additional file 1: SPIRIT 2013 checklist: recommended items to address in a clinical trial protocol and related documents. (DOC 124 kb)

## Appendix

**Table 1:** List of participating research centers as of July 2018

Name of center	Principal investigator
Pitié-Salpêtrière University Hôpital, Paris, France	Martin Dres (National PI France)
Universitätsklinikum Bonn, Germany	Christian Putensen
Universitätsklinikum Carl Gustav Carus Dresden, Germany	Marcelo Gama de Abreu (National PI Germany)
Universitätsmedizin Göttingen, Göttingen, Germany	Onnen Mörer
Herz- und Diabeteszentrum NRW, Bad Oeynhausen, Germany	Christian Flottmann
Charité – Universitätsmedizin Berlin, Germany	Holger Müller-Redetzky
Centre Hospitalier Universitaire, Angers, France	Alain Mercat
Universitätsklinikum Hamburg-Eppendorf Hamburg, Germany	Stefan Kluge
APHP, Hôpital Européean Georges-Pompidou	Jean-Luc Diehl
APHP, Hôpital Louis-Mourier	Jonathan Messika
Universitätsklinikum Jena, Jena, Germany	Andreas Kortgen
Centre Hospitalier Universitaire (CHU) Nouvel Hôpital Civil, Strasbourg, France	Ferhat Meziani
Universitätsklinik RWTH Aachen, Aachen, Germany	Christian Brülls

## Abbreviations

CRO: Contract research organization
eCRF: Electronic case report form
EDC: Electronic data capture
EXdi: Maximal diaphragmatic excursion
ICU: Intensive care unit
ITT: Intent-to-treat population
LCU: Lungpacer Control Unit
MIP: Maximal inspiratory pressure
mITT: Modified intent-to-treat population
MV: Mechanical ventilation
PP: Per protocol population
RSBI: Rapid shallow breathing index
SAE: Serious adverse event
TFdi: Diaphragm thickness and thickening fraction
TTVDP: Temporary transvenous diaphragm pacing
VIDD: Ventilator-induced diaphragmatic dysfunction
VLT: Ventilator liberation trial

## References

[1] Pfuntner A, Wier LM, Stocks C (2013). Most frequent procedures performed in U.S. hospitals, 2011: Statistical Brief #165. Healthcare Cost and Utilization Project (HCUP) Statistical Briefs.

[2] Wunsch H, Kramer A, Gershengorn HB (2017). Validation of intensive care and mechanical ventilation codes in Medicare data. Crit Care Med.

[3] Zilberberg MD, de Wit M, Shorr AF (2012). Accuracy of previous estimates for adult prolonged acute mechanical ventilation volume in 2020: update using 2000-2008 data. Crit Care Med.

[4] Beitler JR, Malhotra A, Thompson BT (2016). Ventilator-induced lung injury. Clin Chest Med.

[5] Slutsky AS, Ranieri VM (2014). Ventilator-induced lung injury. N Engl J Med.

[6] Timsit JF, Esaied W, Neuville M, Bouadma L, Mourvllier B (2017). Update on ventilator-associated pneumonia. F1000Res.

[7] Dres M, Goligher EC, Heunks LMA, Brochard LJ (2017). Critical illness-associated diaphragm weakness. Intensive Care Med.

[8] Jaber S, Jung B, Matecki S, Petrof BJ (2011). Clinical review: ventilator-induced diaphragmatic dysfunction—human studies confirm animal model findings!. Crit Care.

[9] Petrof BJ, Hussain SN (2016). Ventilator-induced diaphragmatic dysfunction: what have we learned?. Curr Opin Crit Care.

[10] Powers SK, Wiggs MP, Sollanek KJ, Smuder AJ (2013). Ventilator-induced diaphragm dysfunction: cause and effect. Am J Physiol Regul Integr Comp Physiol.

[11] Jaber S, Petrof BJ, Jung B, Chanques G, Berthet JP, Rabuel C (2011). Rapidly progressive diaphragmatic weakness and injury during mechanical ventilation in humans. Am J Respir Crit Care Med.

[12] Levine S, Nguyen T, Taylor N, Friscia ME, Budak MT, Rothenberg P (2008). Rapid disuse atrophy of diaphragm fibers in mechanically ventilated humans. N Engl J Med.

[13] Goligher EC, Dres M, Fan E, Rubenfeld GD, Scales DC, Herridge MS (2018). Mechanical ventilation-induced diaphragm atrophy strongly impacts clinical outcomes. Am J Respir Crit Care Med.

[14] Beduneau G, Pham T, Schortgen F, Piquilloud L, Zogheib E, Jonas M (2017). Epidemiology of Weaning Outcome according to a New Definition. The WIND study. Am J Respir Crit Care Med.

[15] Powers SK, DeCramer M, Gayan-Ramirez G, Levine S (2008). Pressure support ventilation attenuates ventilator-induced protein modifications in the diaphragm. Crit Care.

[16] Elkins M, Dentice R (2015). Inspiratory muscle training facilitates weaning from mechanical ventilation among patients in the intensive care unit: a systematic review. J Physiother.

[17] Martin AD, Smith BK, Davenport PD, Harman E, Gonzalez-Rothi RJ, Baz M (2011). Inspiratory muscle strength training improves weaning outcome in failure to wean patients: a randomized trial. Crit Care.

[18] Pavlovic D, Wendt M (2003). Diaphragm pacing during prolonged mechanical ventilation of the lungs could prevent from respiratory muscle fatigue. Med Hypotheses.

[19] Gayan-Ramirez G (2013). Ventilator-induced diaphragm dysfunction: time for (contr)action!. Eur Respir J.

[20] Laghi F, Shaikh H (2014). Preventing ventilator-induced diaphragmatic dysfunction with phrenic nerve stimulation. Crit Care Med.

[21] Le Pimpec-Barthes F, Legras A, Arame A, Pricopi C, Boucherie JC, Badia A (2016). Diaphragm pacing: the state of the art. J Thorac Dis.

[22] Masmoudi H, Coirault C, Demoule A, Mayaux J, Beuvin M, Romero N (2013). Can phrenic stimulation protect the diaphragm from mechanical ventilation-induced damage?. Eur Respir J.

[23] Reynolds SC, Meyyappan R, Thakkar V, Tran BD, Nolette MA, Sadarangani G (2017). Mitigation of ventilator-induced diaphragm atrophy by transvenous phrenic nerve stimulation. Am J Respir Crit Care Med.

[24] Mankowski RT, Ahmed S, Beaver T, Dirain M, Han C, Hess P (2016). Intraoperative hemidiaphragm electrical stimulation reduces oxidative stress and upregulates autophagy in surgery patients undergoing mechanical ventilation: exploratory study. J Transl Med.

[25] Martin AD, Joseph AM, Beaver TM, Smith BK, Martin TD, Berg K (2014). Effect of intermittent phrenic nerve stimulation during cardiothoracic surgery on mitochondrial respiration in the human diaphragm. Crit Care Med.

[26] Ahn B, Beaver T, Martin T, Hess P, Brumback BA, Ahmed S (2014). Phrenic nerve stimulation increases human diaphragm fiber force after cardiothoracic surgery. Am J Respir Crit Care Med.

[27] Adler D, Gottfried SB, Bautin N, Mirkovic T, Schmidt M, Raux M (2011). Repetitive magnetic stimulation of the phrenic nerves for diaphragm conditioning: a normative study of feasibility and optimal settings. Appl Physiol Nutr Metab.

[28] Escher DJ, Ashley W, Ertag W, Parker B, Furman S, Robinson G (1968). Clinical control of respiration by transvenous phrenic pacing. Trans Am Soc Artif Intern Organs.

[29] Costanzo MR, Ponikowski P, Javaheri S, Augostini R, Goldberg L, Holcomb R (2016). Transvenous neurostimulation for central sleep apnoea: a randomised controlled trial. Lancet.

[30] Reynolds S, Ebner A, Meffen T, Thakkar V, Gani M, Taylor K (2017). Diaphragm activation in ventilated patients using a novel transvenous phrenic nerve pacing catheter. Crit Care Med.

[31] Tobin MJ (2012). Extubation and the myth of "minimal ventilator settings". Am J Respir Crit Care Med.

[32] Marini JJ, Smith TC, Lamb V (1986). Estimation of inspiratory muscle strength in mechanically ventilated patients: the measurement of maximal inspiratory pressure. J Crit Care.

[33] Caruso P, Friedrich C, Denari SD, Ruiz SA, Deheinzelin D (1999). The unidirectional valve is the best method to determine maximal inspiratory pressure during weaning. Chest.

[34] Yang KL, Tobin MJ (1991). A prospective study of indexes predicting the outcome of trials of weaning from mechanical ventilation. N Engl J Med.

[35] Ferreira FL, Bota DP, Bross A, Melot C, Vincent JL (2001). Serial evaluation of the SOFA score to predict outcome in critically ill patients. JAMA.

[36] Vincent JL, Moreno R, Takala J, Willatts S, De Mendonca A, Bruining H (1996). The SOFA (Sepsis-related Organ Failure Assessment) score to describe organ dysfunction/failure. On behalf of the Working Group on Sepsis-Related Problems of the European Society of Intensive Care Medicine. Intensive Care Med.

[37] Dube BP, Dres M, Mayaux J, Demiri S, Similowski T, Demoule A (2017). Ultrasound evaluation of diaphragm function in mechanically ventilated patients: comparison to phrenic stimulation and prognostic implications. Thorax.

[38] Goligher EC, Ferguson ND, Brochard LJ (2016). Clinical challenges in mechanical ventilation. Lancet.

[39] Blackwood B, Burns KE, Cardwell CR, O'Halloran P (2014). Protocolized versus non-protocolized weaning for reducing the duration of mechanical ventilation in critically ill adult patients. Cochrane Database Syst Rev.

[40] Rose L, Schultz MJ, Cardwell CR, Jouvet P, McAuley DF, Blackwood B (2015). Automated versus non-automated weaning for reducing the duration of mechanical ventilation for critically ill adults and children: a Cochrane systematic review and meta-analysis. Crit Care.

[41] Gaudry S, Sztrymf B, Sonneville R, Megarbane B, Van Der Meersch G, Vodovar D (2017). Loxapine to control agitation during weaning from mechanical ventilation. Crit Care.

[42] Lai YC, Ruan SY, Huang CT, Kuo PH, Yu CJ (2013). Hemoglobin levels and weaning outcome of mechanical ventilation in difficult-to-wean patients: a retrospective cohort study. PLOS One.

[43] Schonhofer B, Bohrer H, Kohler D (1998). Blood transfusion facilitating difficult weaning from the ventilator. Anaesthesia.

[44] Hetland B, Lindquist R, Chlan LL (2015). The influence of music during mechanical ventilation and weaning from mechanical ventilation: a review. Heart Lung.

[45] Rose L, Adhikari NK, Leasa D, Fergusson DA, McKim D (2017). Cough augmentation techniques for extubation or weaning critically ill patients from mechanical ventilation. Cochrane Database Syst Rev.

[46] Deye N, Lellouche F, Maggiore SM, Taille S, Demoule A, L'Her E (2013). The semi-seated position slightly reduces the effort to breathe during difficult weaning. Intensive Care Med.

[47] Diehl JL, Mercat A, Guerot E, Aissa F, Teboul JL, Richard C (2003). Helium/oxygen mixture reduces the work of breathing at the end of the weaning process in patients with severe chronic obstructive pulmonary disease. Crit Care Med.

[48] Mekontso-Dessap A, de Prost N, Girou E, Braconnier F, Lemaire F, Brun-Buisson C (2006). B-type natriuretic peptide and weaning from mechanical ventilation. Intensive Care Med.

[49] Mekontso Dessap A, Roche-Campo F, Kouatchet A, Tomicic V, Beduneau G, Sonneville R (2012). Natriuretic peptide-driven fluid management during ventilator weaning: a randomized controlled trial. Am J Respir Crit Care Med.

[50] Routsi C, Stanopoulos I, Zakynthinos E, Politis P, Papas V, Zervakis D (2010). Nitroglycerin can facilitate weaning of difficult-to-wean chronic obstructive pulmonary disease patients: a prospective interventional non-randomized study. Crit Care.

[51] Stanopoulos I, Manolakoglou N, Pitsiou G, Trigonis I, Tsiata EA, Boutou AK (2007). Sildenafil may facilitate weaning in mechanically ventilated COPD patients: a report of three cases. Anaesth Intensive Care.

[52] Sterba M, Banerjee A, Mudaliar Y (2008). Prospective observational study of levosimendan and weaning of difficult-to-wean ventilator dependent intensive care patients. Crit Care Resusc.

[53] Dres M, Dube BP, Mayaux J, Delemazure J, Reuter D, Brochard L (2017). Coexistence and impact of limb muscle and diaphragm weakness at time of liberation from mechanical ventilation in medical intensive care unit patients. Am J Respir Crit Care Med.

[54] Carlucci A, Ceriana P, Prinianakis G, Fanfulla F, Colombo R, Nava S (2009). Determinants of weaning success in patients with prolonged mechanical ventilation. Crit Care.

[55] Kim WY, Park SH, Kim WY, Huh JW, Hong SB, Koh Y (2016). Effect of theophylline on ventilator-induced diaphragmatic dysfunction. J Crit Care.

[56] Cader SA, Vale RG, Castro JC, Bacelar SC, Biehl C, Gomes MC (2010). Inspiratory muscle training improves maximal inspiratory pressure and may assist weaning in older intubated patients: a randomised trial. J Physiother..

[57] Condessa RL, Brauner JS, Saul AL, Baptista M, Silva AC, Vieira SR (2013). Inspiratory muscle training did not accelerate weaning from mechanical ventilation but did improve tidal volume and maximal respiratory pressures: a randomised trial. J Physiother..

[58] Vorona S, Sabatini U, Al-Maqbali S, Bertoni M, Dres M, Bissett B (2018). Inspiratory muscle rehabilitation in critically ill adults. A systematic review and meta-analysis. Ann Am Thorac Soc.

[59] Cottereau G, Dres M, Avenel A, Fichet J, Jacobs FM, Prat D (2015). Handgrip strength predicts difficult weaning but not extubation failure in mechanically ventilated subjects. Respir Care.

[60] Dres M, Schmidt M, Ferre A, Mayaux J, Similowski T, Demoule A (2012). Diaphragm electromyographic activity as a predictor of weaning failure. Intensive Care Med.

[61] Jung B, Nougaret S, Conseil M, Coisel Y, Futier E, Chanques G (2014). Sepsis is associated with a preferential diaphragmatic atrophy: a critically ill patient study using tridimensional computed tomography. Anesthesiology.

[62] DiPals Writing Committee, DiPals Study Group Collaborators (2015). Safety and efficacy of diaphragm pacing in patients with respiratory insufficiency due to amyotrophic lateral sclerosis (DiPALS): a multicentre, open-label, randomised controlled trial. Lancet Neurol.

[63] Gonzalez-Bermejo J, Morelot-Panzini C, Tanguy ML, Meininger V, Pradat PF, Lenglet T (2016). Early diaphragm pacing in patients with amyotrophic lateral sclerosis (RespiStimALS): a randomised controlled triple-blind trial. Lancet Neurol.

[64] Ebihara S, Hussain SN, Danialou G, Cho WK, Gottfried SB, Petrof BJ (2002). Mechanical ventilation protects against diaphragm injury in sepsis: interaction of oxidative and mechanical stresses. Am J Respir Crit Care Med.

[65] Le Dinh M, Carreira S, Obert J, Gayan-Ramirez G, Riou B, Beuvin M (2018). Prolonged mechanical ventilation worsens sepsis-induced diaphragmatic dysfunction in the rat. PLOS One.

[66] Demoule A, Divangahi M, Yahiaoui L, Danialou G, Gvozdic D, Petrof BJ (2009). Chemokine receptor and ligand upregulation in the diaphragm during endotoxemia and Pseudomonas lung infection. Mediat Inflamm.

[67] Gonzalez-Bermejo J, Morelot-Panzini C, Georges M, Demoule A, Similowski T (2014). Can diaphragm pacing improve gas exchange? Insights from quadriplegic patients. Eur Respir J.

[68] Masmoudi H, Persichini R, Cecchini J, Delemazure J, Dres M, Mayaux J (2017). Corrective effect of diaphragm pacing on the decrease in cardiac output induced by positive pressure mechanical ventilation in anesthetized sheep. Respir Physiol Neurobiol.

